# Outcomes of IncobotulinumtoxinA Injection on Myalgia and Arthralgia in Patients Undergoing Temporomandibular Joint Arthroscopy: A Randomized Controlled Trial

**DOI:** 10.3390/toxins15060376

**Published:** 2023-06-03

**Authors:** David Faustino Ângelo, David Sanz, Francesco Maffia, Henrique José Cardoso

**Affiliations:** 1 Instituto Português da Face, 1050-227 Lisboa, Portugalhenrique.cardoso@ipface.pt (H.J.C.); 2 Faculdade de Medicina, Universidade de Lisboa, 1649-028 Lisboa, Portugal; 3 Centre for Rapid and Sustainable Product Development, Polytechnic Institute of Leiria, 2430-028 Marinha Grande, Portugal; 4 Clínica Universitária de Estomatologia, Centro Hospitalar Universitário Lisboa Norte-Hospital de Santa Maria, 1649-028 Lisboa, Portugal; 5 Maxillofacial Surgery Unit, Department of Neurosciences, Reproductive and Odontostomatological Sciences, University of Naples “Federico II”, Via Sergio Pansini 5, 80131 Naples, Italy

**Keywords:** Temporomandibular disorder, botulinum toxin, incobotulinumtoxinA, TMJ arthroscopy

## Abstract

Background: Several studies have considered Botulinum Neurotoxin Type A injections effective in treating temporomandibular joint disorder (TMD) symptoms. A double-blind, randomized, controlled clinical trial investigated the benefit of complementary incobotulinumtoxinA (inco-BoNT/A) injections in the masticatory muscles of patients submitted to bilateral temporomandibular joint (TMJ) arthroscopy. Methods: Fifteen patients with TMD and an indication for bilateral TMJ arthroscopy were randomized into inco-BoNT/A (Xeomin, 100 U) or placebo groups (saline solution). Injections were carried out five days before TMJ arthroscopy. The primary outcome variable was a Visual Analogue Scale for TMJ arthralgia, and secondary outcomes were the myalgia degree, maximum mouth opening, and joint clicks. All outcome variables were assessed preoperatively (T0) and postoperatively (T1—week 5; T2—6-month follow-up). Results: At T1, the outcomes in the inco-BoNT/A group were improved, but not significantly more than in the placebo group. At T2, significant improvements in the TMJ arthralgia and myalgia scores were observed in the inco-BoNT/A group compared to the placebo. A higher number of postoperative reinterventions with further TMJ treatments were observed in the placebo group compared to inco-BoNT/A (63% vs. 14%). Conclusions: In patients submitted to TMJ arthroscopy, statistically significant long-term differences were observed between the placebo and inco-BoNT/A groups.

## 1. Introduction

Temporomandibular Joint Disorders (TMDs) are a set of clinical problems affecting the temporomandibular joint (TMJ), surrounding musculature, and associated structures [1]. Following the Diagnostic Criteria for Temporomandibular Disorders (DC/TMD), TMD can be divided into intra-articular joint disorders, masticatory muscle disorders (myalgia), headaches, and pathologies affecting associated structures [2]. The main signs and symptoms of TMD include arthralgia (TMJ pain), myalgia (muscle pain), TMJ sounds (clicking and crepitus), and functional limitations of jaw movements [3].

Due to the complex nature and clinical presentation, TMDs require a multidisciplinary approach and an accurate diagnosis obtained with proper clinical examination and radiological imaging such as magnetic resonance imaging (MRI) and/or computer tomography (CT) scans [4]. Depending on the type and stage of the pathology affecting the TMJ, the treatment can range from less invasive procedures such as TMJ arthrocentesis and TMJ arthroscopy to TMJ open surgery [5]. TMJ arthroscopy is a minimally invasive surgical technique effective in reducing pain and restoring mandibular function with minimal morbidity [1,5]. TMJ arthroscopy is classified into three different levels depending on the operative procedure: Level 1 is considered basic diagnostic arthroscopy with lysis and lavage, level 2 is defined as operative arthroscopy with intra-articular coblation or other surgical techniques, and level 3 arthroscopy implicates a disc suture. When concomitant arthrogenous and muscular disorders are present, there is no scientific accordance with the best therapeutic approach. Almost half of the cases diagnosed with TMD have both articular and muscular components. The combination of both types of the disease is frequently induced by stress-related parafunctional habits (clenching and/or bruxism) [3,6]. Due to its anti-inflammatory and muscle tension-relieving effect, Botulinum Neurotoxin A (BoNT/A) has been studied and applied in TMD [7]. BoNT/A is a neurotoxin produced by bacteria of the genus Clostridium that has two putative effects on the neuromuscular junction: the inhibition of acetylcholine release leading to muscle paralysis and the decrease in the locoregional inflammation by blocking P substance, a neurotransmitter involved in the modulation of the pain perception that binds to specific receptors in lamina I of the spinal cord. Head and neck BoNT/A injections have rare and reversible adverse effects such as dysphagia, muscle weakness, and flu-like symptoms [8]. The beneficial effect of BoNT/A injections has been demonstrated in the published literature; however, there is a heterogeneity of published results about managing persistent pain related to TMD. In a systematic review and meta-analysis, Machado et al. [7] concluded that BoNT/A is more effective than a placebo in pain reduction for TMD patients. Two other systematic reviews demonstrated inconclusive results related to the effectiveness of BoNT/A injections in the treatment of TMD [9,10]. More recently, Al-Moraissi, et al. [11] showed that BoNT/A is a valid option for treating painful TMD of muscular origin. Due to the lack of univocal scientific evidence in the literature, the authors decided to conduct a double-blind, randomized controlled clinical trial with inco-BoNT/A injection in the masticatory muscles as a complementary treatment in patients submitted to bilateral TMJ arthroscopy.

This study aimed to evaluate the possible benefits of injecting inco-BoNT/A in masticatory muscles before TMJ arthroscopy.

## 2. Results

A total of 15 patients with a mean age of 26.5 ± 6.1 years old (range 18–39) were included in the study, resulting in 30 operated joints. All except one patient were females (93.3%) (Table 1). Preoperative arthrogenous TMD diagnoses were (1) arthralgia (*n* = 27 joints); (2) disc displacement without reduction (DDwoR) (*n* = 15 joints); (3) disc displacement with reduction (DDwR) (*n* = 12 joints); (4) osteoarthrosis (*n* = 13 joints); (5) disc perforation (*n* = 1 joint); and (6) osteophytes (*n* = 1 joint) (Table 1). No complications or adverse effects were observed after inco-BoNT/A injections.

The study was designed to have an endpoint at five weeks post-operation. However, an ethical committee preliminary analysis in the study’s middle phase (including 15 patients from the 30 initially planned) found no significant differences between the placebo and inco-BoNT/A groups. Therefore, the ethics committee suggested stopping the study due to an apparent lack of clinical benefit of the drug in the initially designed time frame [12]. The ethical committee also recommended a six-month follow-up to re-evaluate the results. Therefore, based on this recommendation, investigators decided to stop the clinical trial to benefit patients and to perform consecutive analyses at a six-month follow-up.

### 2.1. Pre and Post-Treatment Measurements

Pre- and post-treatment measurements are described in Table 2. Regarding 50% of the included patients, the distribution between the two groups was eight patients (16 joints) in the placebo group and seven (14 joints) in the inco-BoNT/A group.

#### 2.1.1. Pre-Treatment Phase

The mean Visual Analogue Scale (VAS) for TMJ arthralgia before the treatment was 5.7 ± 2.8 (6 ± 3.6 in the placebo group and 5.4 ± 1.9 in the inco-BoNT/A group). The mean MMO was 34.5 ± 11.1 mm (33.9 ± 10.1 mm in the placebo group and 35.1 ± 12.8 mm in the inco-BoNT/A group). The average myalgia degree was 2.6 ± 0.5 (2.8 ± 0.3 in the placebo group and 2.3 ± 0.6 in the inco-BoNT/A group). In addition, joint clicks were assessed, and clicks were found to be present in 13 joints in 8 patients (placebo—8 joints; inco-BoNT/A—5 joints).

#### 2.1.2. Post-Treatment T1 (5 Weeks)

In the postoperative phase, a reduction in VAS for TMJ arthralgia was observed, showing an average of 0.9 ± 1.3–1.6 (0–10) in both groups (*p* = 0.753). MMO increased in both groups (+1.6 mm in the placebo group and +1.2 mm in the inco-BoNT/A), presenting an average MMO of 36.1 ± 2.7 mm (*p* = 0.683). A reduction in the myalgia degree was also observed, with a score of 0.9 ± 1.0 (0–3) in the placebo group and 1.1 ± 1.1 (0–3) in the inco-BoNT/A group (*p* = 0.815). No clicks were observed in the joints of the inco-BoNT/A group, while the placebo group was observed in one patient (right joint).

#### 2.1.3. Post-Treatment T2 (6 Months)

**Figure 1:**
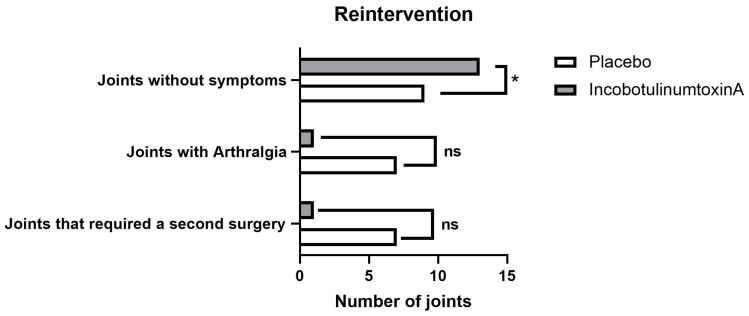
Revaluation six months post-treatment; * *p* ≤ 0.05.

Results from the second clinical evaluation six months post-treatment (T2) are shown in Table 2 and Figure 1. The inco-BoNT/A group presented a lower TMJ arthralgia score and myalgia degree compared to the placebo group (0.14 ± 0.4 vs. 1.3 ± 1.9 and 0.14 ± 0.4 vs. 1.2 ± 1.1 and *p* = 0.036 and *p* = 0.023). A total of eight joints with arthralgia were observed: seven in the placebo group (two bilateral, one from the right joint, and two from the left joint) and one from the inco-BoNT/A group (left joint) (Figure 1). A higher percentage of patients from the inco-BoNT/A group were symptom-free (13 joints—92.9% vs. 9 joints—56.2% in the placebo group) (*p* = 0.007, Figure 1). Eight TMJ patients (27%) required a treatment repetition (seven vs. one joint from the placebo and inco-BoNT/A groups, respectively, Figure 1). Additionally, at the 6-month revaluation, three patients from the placebo group presented persistent myalgia and received inco-BoNT/A treatment at the six-month follow-up.

## 3. Discussion

BoNT/A is a promising therapy used to control myofascial pain and its clinical applications to manage various types of chronic orofacial pain (TMD, secondary headache, and neuralgias) [13]. However, few double-blind, randomized controlled clinical trials in the literature support this approach [9,10,14]. Additionally, the specific mechanisms of chronic pain in somatosensory profiles of BoNT/A are not entirely identified but seem to involve a modulation role in the perception of pain [15].

Five weeks after surgery, our measurements showed a reduction in VAS for TMJ arthralgia and myalgia degree in both groups (Table 2). Furthermore, an increase in the MMO was observed in both groups. Additionally, the total joint clicks were reduced and were only observed in one patient (right joint) in the placebo group. Based on these results at five weeks post-treatment, although some differences between placebo and inco-BoNT/A treatments were observed, these differences among groups were not statistically significant (*p* > 0.05).

It is necessary to consider that TMDs are a complex group of musculoskeletal disorders with a multifactorial etiology. The physical, behavioral, and emotional factors may overlap and interact, resulting in various signs and symptoms. Moreover, the etiology of pain and disability in myofascial pain is complex and reflects an interaction between physical, behavioral, social, and psychological factors, such as higher anxiety levels and depression [16,17,18]. Our results were similar to other studies that did not describe accurate results on the therapeutic benefits of BoNT/A on TMD [9,10]. In a randomized, controlled, double-blind study, Ernberg et al. [14] analyzed the effect of BoNT/A (50 U) for the treatment of myofascial TMD pain compared with placebo (isotonic saline). Although the authors have observed a reduction of pain in the BoNT/A group, the number of patients who experienced a 30% pain reduction was not higher than placebo at any follow-up [15]. In a systematic review by Chen et al. [9], five relevant clinical trials were identified (involving 117 participants), and two trials revealed a significant difference in myofascial pain reduction compared to placebo. Another trial that compared BoNT/A with fascial manipulation showed equal efficacy of pain relief on TMD, while the remaining two trials showed no significant difference between the BoNT/A and placebo groups [8]. Similar results were obtained in the systematic review by Thambar et al. [10], where the authors concluded a lack of consensus on the therapeutic benefit of BoNT/A in managing myofascial TMD. In contrast, other studies injecting the masticatory muscles with BoNT/A suggested a positive role in treating TMD [19]. In a randomized controlled study, Patel et al. [20] used inco-BoNT/A (Xeomin^®^) to evaluate the reduction of stress on the TMJ and the improvement of pain associated with TMJ and muscle disorder [20]. A change in average pain scores was observed four weeks after the first injection with inco-BoNT/A, showing a larger drop in value than the placebo [20]. Kurtoglu et al. [21], in a randomized study that included 24 patients with myofascial pain with or without functional disc displacement, observed that injection of BoNT/A reduced pain and improved psychological status in comparison to the placebo group (saline) after a follow-up period of 14 and 28 days [21]. In a systematic review performed by Ramos-Herrada et al. [22], the analysis of the selected studies has shown that low doses of BoNT/A are effective in treating refractory myofascial pain associated with TMD. However, most of these studies are retrospective and present several methodological issues, such as fewer patients [23].

In our study, statistically significant differences between groups were observed at the six-month follow-up; more patients in the inco-BoNT/A group were symptom-free (9 joints—56.2% vs. 13 joints—92.9% in the placebo and inco-BoNT/A groups, respectively), and fewer patients required a postoperative new TMJ treatment (seven joints—44% vs. one joint—7% in the placebo and inco-BoNT/A groups, respectively). A gradual improvement in the myofascial condition can explain our results. The administration of BoNT/A can potentially result in better long-term outcomes by continuously decreasing tension in the affected muscles and gradually reducing the overload in TMJ. Therefore, a 5-week timeframe might not be sufficient to observe these effects, but they are likely to become apparent later. Similar results were found in the study by Canales et al. [23], where the effects of BoNT/A in the mandibular range of motion and muscle tenderness by palpation were studied in 20 persistent myofascial pain patients. Investigators performed a follow-up at 28 and 180 days after treatment. Regardless of dose, all parameters of the mandibular range of motion significantly improved after 180 days (6 months) in all BoNT/A groups, compared with the control group. Since the pain can constrict to reduce movements, the mandibular range of motion is expected to improve only after significantly reducing muscular tension. Therefore, these results can highlight the importance of pain reduction in patients affected by persistent myalgia to improve mandibular movements quicker and reduce TMJ overload [23].

Currently, there is no consensus on the most appropriate treatment protocol for myofascial pain [24]. In this study, we have selected to treat the masseter and temporalis muscles. However, no defined and precise protocols exist to treat myofascial pain associated with arthrogenous TMD with inco-BoNT/A. Therefore, treating other masticatory muscles could have led to a better outcome, such as those observed in von Lindern JJ et al.’s [25] study where the masseter, temporalis, and medial pterygoid muscles were also injected. Another essential aspect to consider is the duration of the beneficial effects of BoNT/A in the treatment of myalgia. Since the most significant effects of the improvements are seen at six months, further studies are needed to observe their lasting impact on the patient’s clinical condition. Although growing evidence supports the efficacy and safety of BoNT/A in treating myofascial and arthrogenous TMD, most of the published studies have inconclusive results [26]. BoNT/A has been shown to have good efficacy and safety results in other therapeutic indications such as chronic migraines, spasticity, dystonias, and sialorrhea. Therefore, more RCTs with larger sample sizes and longer follow-ups are needed to determine the effectiveness of inco-BoNT/A in the long-term treatment of myofascial pain in patients with TMD [27].

Numerous RCTs published in the literature have been terminated prematurely [28]. In a systematic review, Montori et al. [28] evaluate RCTs’ epidemiology and reporting quality involving interventions stopped early for benefit. The authors identified 143 RCTs stopped early for benefit, of which the majority (92) were published in 5 high-impact medical journals [28].

The limitations of this study need to be addressed. First, the number of patients was small due to the study interruption in the middle. While a larger sample size may not show significant results in the short term (5 weeks), it could confirm the significant improvements in the inco-BoNT/A group observed at six months.

## 4. Conclusions

BoNT/A improved the outcomes as a complementary treatment in patients who were candidates for TMJ arthroscopy, reducing arthralgia and myalgia in the long term. This study did not accomplish the primary objective since inco-BoNT/A and placebo groups were not statistically significantly different after five weeks (*p* > 0.05). However, long-term significant differences (*p* < 0.05) related to myalgia degree and articular pain were observed between the placebo and inco-BoNT/A groups. The inco-BoNT/A group also experienced a reduced incidence of post-treatment persistent symptoms and the subsequent necessity of further treatments. Consequently, in this randomized controlled clinical trial, inco-BoNT/A treatment has been shown to play a crucial role in reducing the need for TMJ reinterventions and the score of arthralgia and myalgia in the long term. In the near future, further studies are necessary to evaluate the long-term effects in a larger cohort.

## 5. Materials and Methods

### 5.1. Study Design

A double-blind, randomized, controlled clinical trial was conducted between January 2021 and December 2021. This study was approved by the Ethics Committee of the Portuguese Institute of the Face (PT/IPFace/RCT/0121/07). The study was registered in ClinicalTrials.Gov (NCT04810429). All the patients signed the informed consent in accordance with the current legislation and the guidelines of the Declaration of Helsinki.

Patients of both genders with TMD diagnosis and indication for bilateral TMJ arthroscopy were recruited. The inclusion criteria included: (1) Age of 18–60 years; (2) history of conservative treatment for TMD without significant improvement for at least three months; (3) clinical diagnosis of bilateral arthrogenous disorder; (4) clinical diagnosis of myalgia; (5) clinical criteria for bilateral TMJ arthroscopy (Dimitroulis Classification 2–4) [29]; (6) magnetic resonance imaging (MRI) and computer tomography (CT) scan documenting the arthrogenous TMD. Subjects who presented any of the following criteria were not eligible for the study: (1) Pregnancy; (2) lactation; (3) previous mandibular trauma; (4) other previous TMD treatment; (5) previous facial treatment with BoNT/A; and (6) any contraindication to the use of BoNT/A according to the XEOMIN prescribing information.

### 5.2. Outcomes Evaluation

All the outcomes were assessed preoperatively (T0) and postoperatively (T1 at 5 weeks and T2 at the 6-month follow-up). T2 was a further evaluation of the treated patients and was initially not included in the study design. The goal was to assess whether the long-term effects were maintained after T1.

The primary outcome was the level of TMJ arthralgia (VAS, 0–10). Arthralgia was reported if it was verified according to (1) a history of pain in the TMJ area and (2) pain modified with jaw movement, function, or parafunction [2,30].

The secondary outcomes were myalgia degree (from G0 to G3), TMJ clicking, and maximum mouth opening (MMO, mm). MMO was measured using a certified ruler between the incisor’s teeth. Myalgia was diagnosed according to a bilateral clinical examination by palpation of temporomandibular muscles, and clinical history positive for (1) pain in the jaw, in front or inside the ear during the past 30 days, and the examiner confirming the masticatory muscles as a source of pain, (2) pain modified with jaw movement, function, or parafunction and a positive clinical evaluation for palpation pressure (5 s/1 kg pressure) in masseter and temporalis muscles as defined in DC/TMD [2,30]. The final arthrogenous diagnosis (including disc displacement with/without reduction, disc perforation, and osteoarthrosis) was confirmed using a CT scan and MRI.

### 5.3. Myalgia and Arthralgia Assessment

The level of TMJ arthralgia was registered through pain during palpation of the lateral or around the lateral pole or pain on maximum unassisted or assisted opening, right or left lateral movements, or protrusive movements, following the DC/TMD guidelines [2,30]. The patient scored pain (0–10) during the palpation test.

Myalgia was graded according to pain intensity in the left and right masseter and temporalis muscles: 0 = No Pain/Pressure Only; 1 = Mild Pain; 2 = Moderate Pain; 3 = Severe Pain [31].

### 5.4. Randomization

Subjects were randomly assigned to two groups: Placebo (NaCl saline solution, excluding human serum albumin and sucrose) or inco-BoNT/A (Xeomin^®^) groups. For this allocation, groups were prepared with a code previously distributed to the two groups. Five days before the TMJ arthroscopy, each patient was booked for a preoperative consultation to perform the blind injection. Treatment assignment was conducted by an independent person, not involved in performing any other procedures in the study such as the preoperative injection or the TMJ arthroscopy. An envelope containing a code was selected and assigned to the patient. Subjects and investigators were blinded to inco-BoNT/A and placebo assignments. The surgeon (D.F.A) was unblinded after all assessments were completed.

### 5.5. Treatment Protocol

#### 5.5.1. Masticatory Muscles Injection

The injection of inco-BoNT/A or 0.9% NaCl saline solution (NaCl, 308 mOsm/L or 9.0 g per liter, B. Braun, Melsungen, Hessen, Germany) was carried out 5 days before the TMJ arthroscopy. The administrations were carried out as follows: The inco-BoNT/A dose was 100 U distributed in 2 syringes of 1 mL each: 25 U (0.5 mL) in each masseter muscle (right and left) and 25 U (0.5 mL) in each temporal muscle (right and left). A single dose of 100 U of BoNT/A has been shown to be effective and without adverse effects [21,32,33]. Guarda-Nardini, et al. [33], in a preliminary randomized clinical trial, demonstrated the efficacy of BoNT/A for reducing myofascial pain with intramuscular injections to each side in the masseter muscles (30 U) and in the anterior temporalis muscles (20 U), for a total treatment of 100 U. The 0.9% NaCl saline solution was injected similarly (2 syringes of 1 mL each): 0.5 mL was distributed in each masseter muscle (right and left), and the procedure was repeated for the right and left temporal muscles. The injection technique was the same for both groups: using a 31 G × 12 mm needle, muscles were gently palpated and then injected, following the dose previously described. The injection depth was detected by pricking the muscle with the needle and then by injecting the placebo or BoNT/A into a more superficial layer, ensuring always staying in the belly of the muscle. All injection guidelines were respected in accordance with anatomical aspects [34].

#### 5.5.2. TMJ Arthroscopy

Five days later, TMJ arthroscopy was performed using a 1.9-mm arthroscope, including a video system (Stryker, San Jose, CA, USA), with a 2.8-mm outer protective cannula. Additional equipment included a surgical scalpel (#11 blade), infusion tube, three-way pipe, 21-G needle, and ringer solution as part of the armamentarium [5]. All the procedures were Level 2 (double portal) TMJ arthroscopies. The standard puncture with an entry point 10 mm anterior and 2 mm below the Holmlund–Hellsing (H-H) line was used to perform the first portal access. The arthroscope was inserted into the superior joint space. A second puncture with a 21-G needle was performed 30 mm anterior and 7 mm below the H-H line to wash the joint with 250–300 mL Ringer solution. The second portal was performed using a triangulation technique, approximately 30–40 mm anterior and 8–12 mm below the H-H. Then, different techniques were used in all patients: intrasynovial medication, tissue coblation with the ReFlex Ultra 45 Plasma Wand system, and capsulotomy. After washing the joint, 1.5–2 cc of hyaluronic acid was injected. All the TMJ arthroscopies performed were level 2 and were completed successfully without complications. Antibiotic protocol (amoxicillin/clavulanic acid or clarithromycin) and non-steroidal anti-inflammatory drugs (ibuprofen) were routinely prescribed following surgery. All patients were instructed to follow a soft diet for three days after intervention and five physiotherapy and three speech therapy sessions starting 3–5 days after the intervention.

### 5.6. Statistical Analysis

All statistical procedures were performed with IBM SPSS Statistics 26.0.0 (IBM Co., Armonk, New York, NY, USA) software. Numerical variables were expressed as the mean (±standard deviation). Normality was determined using the Shapiro-Wilk test. Descriptive analysis was analyzed through Fisher’s exact test. Pre- and post-treatment measurements were first analyzed with non-parametric Friedman’s test (numerical variables) and Cochran’s Q test (binary variables) for each group. Then, the two groups were compared with the non-parametric Mann–Whitney U test for each checkpoint. The statistical significance threshold was *p* < 0.05.

## Figures and Tables

**Table 1:** Baseline characteristics of patients.

	Total (*n* = 15) (30 Joints)	Placebo (*n* = 8) (16 Joints)	Incobotulinumtoxin A (*n* = 7) (14 Joints)	*p* Value
Demographic data				
Age, A ± SD	26.5 ± 6.1 (18–39)	29.3 ± 5.4 (24–39)	23.4 ± 5.5 (18–30)	0.058
Sex (F)	93.3% (14)	100% (8)	85.7% (7)	0.467
Joint Diagnosis T (B, R, L)				
DDwoR	15 (5, 8, 7)	8 (3, 4, 4)	7 (2, 4, 3)	0.694
DDwR	12 (2, 5, 7)	6 (2, 3, 3)	6 (1, 2, 4)	0.417
Osteoarthrosis	13 (4, 7, 6)	5 (2, 2, 3)	8 (2, 5, 3)	0.999
Arthralgia	27 (13, 14, 13)	14 (7, 7, 7)	13 (6, 7, 6)	0.457
Disc Perforation	1 (0, 0, 1)	0 (0, 0, 0)	1 (0, 0, 1)	0.999
Osteophytes	1 (0, 1, 0)	1 (0, 1, 0)	0 (0, 0, 0)	0.999

F: Female; DDwoR: Disc displacement without reduction; DDwR: Disc displacement with reduction. B: Bilateral; R: Right; L: Left; T: Total; A: Average; SD: Standard deviation.

**Table 2:** Pre- and post-treatment measurements.

	Placebo (*n* = 8) (16 Joints)	Inco-BoNT/A (*n* = 7) (14 Joints)	Placebo vs. Inco-BoNT/A *p* Value
Pre-treatment measurements			
TMJ arthralgia (VAS, 0–10), A ± SD	6 ± 3.6	5.4 ± 1.9	0.487
MMO (mm), A ± SD	33.9 ± 10.1	35.1 ± 12.8	0.953
Myalgia degree (0–3), A ± SD	2.8 ± 0.3	2.3 ± 0.6	0.072
Joint clicks T (B,R,L)	8 (4, 4, 4)	5 (1, 2, 3)	0.484
Post-treatment measurements (5 weeks)			
TMJ arthralgia (VAS, 0–10), A ± SD	0.9 ± 1.6	0.9 ± 1.3	0.753
MMO (mm), A ± SD	35.9 ± 2.2	36.3 ± 3.3	0.683
Myalgia degree (0–3), A ± SD	0.9 ± 1.0	1.1 ± 1.1	0.815
Joint clicks T (B,R,L)	1 (0, 1, 0)	0 (0, 0, 0)	0.999
Post-treatment measurements (6 months)			
TMJ arthralgia (VAS, 0–10), A ± SD	2.4 ± 2.1	0.14 ± 0.4	0.036 *
MMO (mm), A ± SD	39.9 ± 4.4	41.0 ± 3.9	0.728
Myalgia degree (0–3), A ± SD	1.2 ± 1.1	0.14 ± 0.4	0.023 *
Joint clicks T (B,R,L)	3 (0, 1, 2)	3 (1,1,0)	0.999
Intragroup analysis (*p*-value)			
TMJ arthralgia	0.055	0.002 **	
MMO	0.079	0.368	
Myalgia degree	0.002 **	0.003 **	
Joint clicks	0.020 *	0.042 *	

VAS: Visual Analogue Scale; MMO: Maximum mouth opening; TMJ: Temporomandibular joint; T: Total; mm: Millimeters VAS scale was 0 indicates no pain and 10 pain as bad as it could be; A: Average; SD: Standard deviation; * *p* ≤ 0.05; ** *p*≤ 0.01.

## Data Availability

The data presented in this study are available in this article.
